# Selected Alien Macroalgae Species from Madeira Archipelago as a Source of Sustainable Antifungal and Elicitor Agents: A Review on Their Valorization Potential and Green Extraction Approaches

**DOI:** 10.3390/md24060206

**Published:** 2026-06-10

**Authors:** Emmanuel Nunes, Nuno Nunes, Miguel Â. A. Pinheiro de Carvalho

**Affiliations:** 1 ISOPlexis Centre for Sustainable Agriculture and Food Technology, Campus da Penteada, University of Madeira, 9020-105 Funchal, Portugal; emmanuel.nunes@staff.uma.pt (E.N.); miguel.carvalho@staff.uma.pt (M.Â.A.P.d.C.); 2 Centre for the Research and Technology of Agroenvironmental and Biological Sciences (CITAB), Inov4Agro, Universidade de Trás-os-Montes e Alto Douro (UTAD), Quinta de Prados, 5000-801 Vila Real, Portugal; 3 Faculty of Life Sciences, Campus da Penteada, University of Madeira, 9020-105 Funchal, Portugal

**Keywords:** non-indigenous macroalgae, non-native macroalgae, beach-cast macroalgae, *Botrytis cinerea*, natural elicitor, natural deep eutectic solvents

## Abstract

Non-indigenous or alien macroalgae are increasingly recognized as ecological threats, sources of raw material, and reservoirs of bioactive compounds for industry and agriculture. This review analyses the valorization potential of this biomass, focusing on their antifungal and elicitor activities against phytopathogenic fungi, particularly Mediterranean (De Bary) Whetzel, 1945. The literature published since 2020 was retrieved from Scopus using targeted keyword combinations. Three major topics were examined: (i) invasive and beach-cast macroalgal and their ecological context, (ii) antifungal and elicitor properties of macroalgal extracts, and (iii) the use of deep eutectic solvents (DES) for the green extraction of bioactive compounds. Species such as *Asparagopsis armata*, *Rugulopteryx okamurae*, and *Sargassum muticum* have shown promising antifungal and elicitor effects, frequently associated with phenolic compounds and polysaccharides. Extracts from these algae can inhibit the growth of fungi or activate plant defense pathways, providing environmentally friendly alternatives to synthetic pesticides. Moreover, DES and natural DES (NADES) offer tunable, biodegradable solvents capable of efficiently extracting these bioactive molecules while reducing the environmental impact associated with conventional organic solvents. Overall, the valorization of this biomass represents a sustainable strategy that simultaneously mitigates ecological and economic impacts and contributes to the development of sustainable inputs in agriculture.

## 1. Introduction

Non-indigenous species (NIS), also known as alien, non-native, foreign or exotic species [1], are organisms introduced into new geographical areas, where they survive and reproduce [2]. The introduction of NIS is mainly a result of human activity, either deliberately or accidentally [3], and this phenomenon is increasing worldwide due to market globalization and tourism [2,4]. Most NIS persist without significantly altering native ecosystems, and many have even been used for agriculture, forestry, and aquaculture purposes [4,5,6,7]. However, some NIS exhibit rapid growth and become invasive when exposed to certain conditions [8,9], with ecologic and economic consequences [2,7]. These invasive species are a major cause of biodiversity loss worldwide as they displace native species and disrupt the structure and functioning of ecosystems [10].

The impact of invasive species is particularly concerning in marine environments. These are highly biodiverse and complex habitats that host a vast array of macro- and microorganisms, which ensure important ecological processes. However, such habitats are continuously threatened by human activity and climate change. These factors can disrupt the natural barriers between marine ecosystems, facilitating the proliferation of invasive species [3,11,12] and contributing to a steady increase in NIS introductions worldwide [13], with shipping transport as the major vector [14,15]. In the European Union, the number of marine NIS increased between 2000 and 2020, with an average of 21 introductions per year [10], and some have displayed invasive behavior [16]. Most marine NIS in Europe are native to the Indo-Pacific Ocean, with special relevance for the Western Indo-Pacific [13,15]. When they become invasive, these species pose an ecological threat and interfere with fishing, aquaculture, and tourism, which are major economic activities in coastal countries [17].

Non-indigenous macroalgae and excessive beach-cast accumulations are an ecological disturbance for marine environments. Their removal is the main strategy to prevent further ecological damage, but this is a costly process. However, considering the biotechnological potential of macroalgae, which remains underexplored, such biomass can be valorized for sustainable extraction and agricultural applications, mitigating ecological and economic impacts. In comparison with native macroalgae, invasive and beach-cast species are particularly attractive candidates for sustainable bioproduct development due to their high biomass availability, rapid growth rates, and low acquisition cost. Unlike the harvesting of native species, which may raise ecological sustainability concerns, the removal of invasive macroalgae is often required as part of ecosystem management strategies. Consequently, biomass collection can simultaneously contribute to environmental protection and resource recovery within circular bioeconomy frameworks. Furthermore, many invasive macroalgae contain high concentrations of bioactive compounds, including phenolics, polysaccharides, terpenoids, and halogenated metabolites, which have demonstrated antioxidant, antimicrobial, antifungal, and elicitor-related properties with potential applications in agriculture and biotechnology [8,18]. Transforming this biomass into a source of bioactive compounds with antifungal and elicitor properties could bridge biodiversity management and plant protection strategies in a key step towards a sustainable economy.

This review aims to provide a comprehensive overview of the current knowledge on non-indigenous macroalgae and beach-cast macroalgae potential, by focusing on green extraction technologies (DES/NADES) and the bioactivity of their extracts against *Botrytis cinerea* (De Bary) Whetzel, 1945 and other phytopathogenic agents. It integrates ecological, technological, and applied perspectives to highlight sustainable biomass recovery strategies and circular bioeconomy development. The review synthesizes peer-reviewed literature with particular attention to studies addressing extraction efficiency, chemical composition, antifungal efficacy, and elicitor potential.

## 2. Bibliographic Analysis

**Figure 1:**
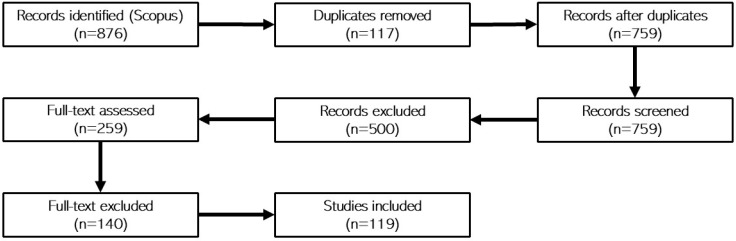
Flow diagram of the literature selection process used in this review. A total of 876 records were initially identified through Scopus database searches. Ultimately, 119 studies were included in the final analysis.

A comprehensive literature search was conducted using the Scopus database to identify relevant studies on macroalgae and their potential applications in plant protection. Searches were performed in the TITLE-ABS-KEY fields and limited to articles and reviews published in English from 2020 onwards. To ensure broad coverage, a high-sensitivity search strategy was applied using three main thematic queries. The first query focused on the occurrence of invasive and non-indigenous macroalgae, using the terms (macroalgae OR seaweed OR “marine algae”) AND (invasive OR “non-indigenous” OR “non-native” OR alien), which retrieved 641 records. The second query addressed the bioactivity of macroalgal extracts, particularly antifungal and elicitor properties, using the terms (macroalgae OR seaweed OR “marine algae”) AND (extract* OR “bioactive compound*” OR “natural product*”) AND (antifungal OR antimicrobial OR elicitor OR “plant defense” OR “biocontrol”) AND (phytopathogen* OR “plant pathogen*” OR fungus OR fungi OR “*Botrytis cinerea*”), yielding 160 records. The third query focused on green extraction technologies, specifically deep eutectic solvents (DES/NADES), using the terms (macroalgae OR seaweed OR “marine algae”) AND (“deep eutectic solvent*” OR DES OR NADES OR “green solvent*”) AND (extraction OR recovery OR “bioactive compound*”), retrieving 75 records. In total, 876 records were identified (Figure 1). All retrieved references were exported to Mendeley Reference Manager, where 117 duplicate entries were removed. As the search queries were not mutually exclusive, overlapping records were expected and addressed during the deduplication step. The remaining 759 records were screened based on titles and abstracts, of which 500 were excluded due to lack of relevance to the scope of the study. A total of 259 articles were assessed for full-text eligibility. Of these, 140 were excluded for the following reasons: lack of relevance to plant protection applications (n = 52), absence of antifungal or elicitor activity data (n = 38), studies not involving macroalgae (n = 21), focus on unrelated applications such as biofuels, cosmetics or nanotechnology (n = 17), and insufficient methodological detail or inaccessible full text (n = 12). Ultimately, 119 studies were included in the final analysis. Restrictive filters such as specific species names or geographic locations were intentionally avoided during the initial search phase to maximize sensitivity and prevent the exclusion of relevant studies. Instead, these aspects were considered during the screening and data extraction stages. Titles and abstracts were screened for relevance, followed by full-text assessment based on predefined inclusion and exclusion criteria. Additionally, a snowballing approach was applied by screening the references of selected articles to identify further relevant studies. Bibliographic data were managed using Mendeley Reference Manager and Mendeley Desktop (v1.19.8).

## 3. Ecological and Economic Impacts of Invasive and Beach-Cast Macroalgae

Macroalgae, also known as seaweeds, are macroscopic photosynthetic organisms ubiquitous in marine environments and are classified into three distinct groups based on their pigmentation: green algae (Chlorophyta), red algae (Rhodophyta), and brown algae (Phaeophyceae) [19,20]. They are quite diverse in terms of morphology and function, with approximately 10,000 species worldwide [21]. They play key ecological roles in coastal ecosystems by contributing to primary production, providing essential nutrients and energy for aquatic organisms [22]. Furthermore, they are known for their extensive applications, including nutraceutical, pharmaceutical, and agricultural applications [21,23].

Non-indigenous macroalgae, however, pose a particular threat to marine environments. Many of them have become invasive, replacing native species and altering the structure of local ecosystems. The expansion of these macroalgae in new habitats depends on several factors. An important one is their biology [24]. Macroalgal biology determines growth rates, life cycles, recruitment rates, physiology, size, and fitness. Invasive algae display high growth rates, dynamic life cycles and high recruitment rates compared to native species [25,26]. Invasive algae are also tolerant to a wide range of temperature and salinity conditions, which further contributes to their expansion in host communities. The invasion mechanism, however, remains mainly unknown due to their complexity [25]. Other factors that contribute to invasion success include the number and frequency of introductions into the new area, as well as the susceptibility of the host community to invasion [24]. Additionally, the excess of nutrients in marine habitats from urban and agricultural activities may trigger invasive traits of non-indigenous macroalgae. Abiotic factors, such as temperature, CO_2_, and light, also induce invasive traits [8]. Understanding such factors is important for the management of these species, which we aim to prevent ecological and economic damage.

Beach-cast macroalgae refers to the accumulation of macroalgae along beach shores, where it settles as beach wrack [27]. These depositions may be composed of native or non-indigenous species that naturally detach from the sea bottom and accumulate on the shore, or can be drift macroalgae from exogenous habitats, carried by currents and winds and finally deposited on the coastlines [28]. This biomass provides organic matter and nutrients for local plants and local invertebrate communities in the beach ecosystem [20,27]. However, an excessive influx of beach-cast macroalgae, often caused by climate changes or anthropogenic activities, has several consequences [20]. It interferes with fisheries and tourism due to the strong odors released during decomposition, affecting the local economy [29]. It also creates anoxic/hypoxic conditions, killing multiple native organisms and disrupting the local ecosystem [28,30].

Invasive and beach-cast macroalgae is a global concern. In the European Union (EU), invasive species are regulated under Regulation (EU) No 1143/2014, which aims to prevent the introduction and mitigate the impact of non-indigenous species. This list was updated in 2022 by Regulation (EU) 2022/1203, with the incorporation of 22 new invasive species, including one macroalgal species [31]. This regulation, however, does not fully address the impact of invasive species in the coastal environments of the EU [15], as only a small number of marine species are included in the Union list [32]. Previous studies have reported the presence of non-indigenous macroalgae in several European coastal regions, such as the Mediterranean [33,34] and the Iberian Peninsula [25]. In Portugal, harmful macroalgal blooms in the south were caused by the invasive species *Asparagopsis armata* (Rhodophyta), *Rugulopteryx okamurae* (Phaeophyceae), and the autochthonous *Ulva* sp. (Chlorophyta), each with unique spatial distribution patterns [8]. In the Azores archipelago, an inventory of non-indigenous macroalgae included the invasive algae *Asparagopsis taxiformis*, *A. armata*, and *R. okamurae* [14]. Ultimately, Pereira et al. (2024) [1] provides a comprehensive overview of the diversity, distribution, introduction pathways, and ecological impacts of non-indigenous macroalgae in the Iberian Peninsula, highlighting their rapid spread and implications for native ecosystems and coastal management.

## 4. Case Studies: Non-Indigenous and Beach-Cast Macroalgal Species Located in Madeira Archipelago

The Madeira archipelago, in the North Atlantic Ocean, includes the inhabited islands of Madeira and Porto Santo, as well as the uninhabited Desertas and Selvagens islands. These latter are Marine Protected Areas (MPAs) [35]. The archipelago, particularly the MPAs, is vulnerable to marine biological invasions due to the strong maritime traffic and tourism, which are important vectors for NIS. The absence of monitoring and early detection programs further increases the archipelago’s susceptibility to marine NIS [36]. Also, the steady increase in anthropogenic activities on the island of Madeira, such as coastal urbanization and tourism, has led to a decline in native macroalgae of the family Sargassaceae, making local ecosystems more susceptible to the spread of NIS macroalgae [37]. As of 2023, 19 macroalgae species are officially recognized as invasive in the Madeira archipelago: *Anotrichium* cf. *okamurae* Baldock, *Anotrichium furcellatum* (J. Agardh), *Antithamnion amphigeneum* A.J.K.Millar, *Antithamnion densum* (Surh) M.A. Howe, *Antithamnion nipponicum* Yamada et Inagaki, *Antithamnionella spirographidis* (Schiffner) E. M. Wollaston, *Antithamnionella ternifolia* (J.D. Hooker and Harvey) Lyle, *Asparagopsis armata* Harvey, *Caulerpa taxifolia* (M.Vahl) C.Agardh, *Colpomenia peregrina* Sauvageau, *Dasya sessilis* Yamada, *Gambierdiscus excentricus* S.Fraga, *Gracilaria vermiculophylla* (Ohmi) Papenfuss*, Grateloupia turuturu* Yamada, *Gymnodinium catenatum* Graham, *Ostreopsis* cf. *ovata* Fukuyo*, Sargassum muticum* (Yendo) Fensholt, *Symphyocladia marchantioides* (Harvey) Falkenberg, and *Undaria pinnatifida* (Harvey) Suringar [38]. Although not officially reported, a study has documented the invasive algae *R. okamurae* in the archipelago [39]. NIS macroalgal blooms pose a threat to the economy of the Madeira archipelago, which relies on tourism and fisheries. The archipelago, however, has important agriculture and viticulture sectors, where this biomass can be used as biostimulant, biofertilizer or biopesticide. This contributes to biomass valorization and mitigates the costs associated with its removal from the shores [28], while simultaneously protecting local ecosystems [3]. Here, we focus on the description of some non-indigenous macroalgae in the Madeira Archipelago, including *A. armata* (Rhodophyta), *Sargassum muticum* (Phaeophyceae) [38], and *R. okamurae* [39].

### 4.1. Asparagopsis armata

*Asparagopsis armata* Harvey 1855, a red macroalga (phylum Rhodophyta) native to Australia and New Zealand, was first described by the botanist William H. Harvey in 1854 [40,41]. *A. armata* was first introduced in Europe in the 1920s through the Mediterranean Sea and the Atlantic [5]. Among other European countries, it is spread in Portugal, including mainland [8], Azores [42] and Madeira [43] archipelagos, where it is considered invasive. *A. armata* can rapidly spread in recipient ecosystems and become the dominant species, displacing native alga and introducing ecological changes that threaten the local fauna [44,45]. In a previous study carried out in the Azores, the presence of *A. armata* in rocky intertidal pools was associated with reduced macroalgal species richness, diversity and variability, resulting in a more homogeneous taxonomic structure. In contrast, non-invaded pools showed higher richness and greater variability in macroalgal taxonomic composition [5]. Additionally, the native red alga *Ellisolandia elongata* was significantly affected by the presence of *A. armata* in invaded zones [5]. Another study, also conducted in the Azores, showed a similar outcome by reporting a negative correlation between the presence of *A. armata* and the diversity of native algal species [24]. The study also found a negative correlation with *Asparagopsis taxiformis*, suggesting that both algae compete [24]. *A. armata* also harms local fauna. In a study, exudates from this alga affected the oxidative stress status and neurophysiology of native mussel *Mytilus galloprovincialis*, with increased toxicity under warm conditions [46]. These studies highlight the invasive potential and ecological threat posed by the alga *A. armata*. It can displace native algal species and threaten native fauna, ultimately altering local ecosystems. Given its invasive potential, further studies are needed to assess the impact of *A. armata* in different regions. In the case of the archipelago of Madeira, no studies on the spatial distribution and the ecological impact of the algae are available.

### 4.2. Rugulopteryx okamurae

*Rugulopteryx okamurae* (E.Y. Dawson) I.K. Hwang, W.J. Lee and H.S. Kim 2009 is a brown macroalgae native to the northwest Pacific Ocean [47]. The alga was recently incorporated into the Regulation (EU) 2022/1203 as an invasive species in the EU [48]. Its occurrence in Europe was first documented in 2002 in the Thau lagoon (Mediterranean, France) without displaying invasive behavior [39,49]. However, massive blooms of the alga were recorded in the Strait of Gibraltar, in Ceuta, between 2015 and 2016 [50,51], and later in France [52], Italy [53,54], and Morocco [55]. *R. okamurae* is also present in Portugal in the continental and insular regions [8,39,56]. The alga, therefore, rapidly spread along the EU’s Atlantic and Mediterranean coasts of the EU between 2017 and 2019, starting from the south of the Iberian Peninsula [57].

*R. okamurae* has a strong invasive capacity, colonizing established communities and displacing native species in a very short time [57]. In Ceuta and the south of the Iberian Peninsula, the alga reached coverage rates of 90–100% at depths between 0 and 20 m, displacing native benthic communities [57,58]. A similar situation was reported in the Calanques National Park, France. Here, the arrival of *R. okamurae* has altered the ecosystem structure, with a decline in biodiversity and the richness of both fauna and algal populations. Clear differences in benthic communities between invaded and non-invaded locations were reported. No distinction among invaded sites was observed, which suggests a homogenization effect [59]. Additionally, in those regions, *R. okamurae* persists throughout the year despite the variation in environmental conditions, further demonstrating its invasive capacity [59,60]. In the Azores archipelago, where *R. okamurae* was first detected in 2019, the alga has become the dominant species in some locations, reaching 100% coverage rates in both sun exposed and shaded rock surfaces [56]. In continental Portugal, the incidence of *R. okamurae* increased from 2021 to 2023, even surpassing other invasive species such as *A. armata*, and it is continuously expanding along the Atlantic coast of the country [8]. In the Madeira Archipelago, *R. okamurae* is not officially listed among the invasive species, although its arrival to the Madeira Island has already been reported by Bernal–Ibáñez et al. (2022) [38]. Here, the alga poses a medium-to-high risk of becoming invasive, with a strong possibility of colonizing hard substrates. However, according to the authors, the possibility of massive blooms by *R. okamurae* under the climatic conditions of the island is uncertain. These studies demonstrate the strong invasive capacity of *R. okamurae*, which can colonize different habitats and replace entire communities in a short time. This poses a threat to local ecosystems and socio-economic activities, highlighting the need for urgent mitigation strategies.

### 4.3. Sargassum *sp*.

*Sargassum* is a genus of brown macroalgae of the family Sargassaceae, order Fucales. It comprises over 350 species distributed across temperate, subtropical, and tropical regions [61]. Approximately 60 species occur in the Atlantic Ocean [62], some with invasive behavior [3]. *Sargassum* species display distinct ecological behaviors and invasion pathways. In this review, two different situations are considered: (i) pelagic species, namely *Sargassum fluitans* and *Sargassum natans*, which are associated with large-scale drifting biomass accumulations and beach-cast events, and (ii) the invasive benthic species *Sargassum muticum*, which has become established in several European coastal ecosystems, including the Madeira Archipelago. Although ecologically distinct, both scenarios generate substantial biomass for valorization.

#### 4.3.1. Pelagic *Sargassum* Species (*S. fluitans* and *S. natans*)

Excessive accumulations of pelagic *Sargassum* spp. along the coasts has become an ecological concern in many regions, such as of the Gulf of Mexico, the Atlantic, the Caribbean and West Africa [63]. In 2018, a massive incursion of pelagic *Sargassum* spp. along the Mexican Caribbean coast led to the death of 75 different animal species. The products of biomass decomposition include ammonium and hydrogen sulfide, which create hypoxic conditions in water and kill the fauna [30]. *Sargassum* depositions were also registered in the Azores and the Madeira archipelagos between 2023 and 2024, with no major ecological impacts. The biomass was composed of *Sargassum fluitans* and *Sargassum natans*, non-native species, and was accompanied by non-native epiphytic fauna that could represent a threat to local biodiversity [62,63].

#### 4.3.2. *Sargassum muticum*

Another case of concern is *Sargassum muticum* (Yendo) Fensholt, an alga species native to Japan and introduced to Europe in the 1970s [64]. It is present in several European regions, including the Iberian Peninsula [25], Azores and Madeira archipelagos [17,38]. The invasive behavior of this alga has been demonstrated. The presence of *S. muticum* showed a negative impact on *Fucus vesiculosus* and *Cladostephus spongiosus*, which are native macroalgae species in Lough Hyne, Ireland [65]. Another study demonstrated that *S. muticum* exhibits greater antifouling capacity than native *Sargassum* species, likely due to toxic secondary metabolites that contribute to its invasive success [66].

### 4.4. Valorization Potential of Invasive Macroalgae

Beyond their ecological implications, the increasing accumulation of invasive and beach-cast macroalgae also creates opportunities for biomass valorization. Their rich biochemical composition, including polysaccharides, phenolics, and pigments, supports the development of sustainable applications in agriculture, biotechnology, and bio-based industries.

Importantly, not all invasive macroalgae exhibit the same valorization potential. Red macroalgae such as *Asparagopsis armata* are particularly recognized for their rich content of halogenated metabolites, which are frequently associated with direct antimicrobial and antifungal activity [67]. In contrast, brown macroalgae including *Rugulopteryx okamurae* and *Sargassum muticum* are generally richer in phlorotannins and sulfated polysaccharides, compounds often linked to antioxidant and elicitor-related activities [68]. Green macroalgae such as *Ulva* spp. are mainly valued for their ulvan-rich polysaccharide fractions, which have shown promising plant defense elicitation properties [69]. These biochemical differences strongly influence both extraction selectivity and the resulting biological activities, highlighting the importance of species-specific valorization strategies.

## 5. Macroalgae Valorization and Potential Applications

Massive depositions of exotic beach-cast macroalgae are an ecological and economic threat [28,30,63]. Large accumulations of algae in the Caribbean have led to massive holiday cancelations along with negative media coverage, strongly affecting the tourism activity in the region [70,71]. The main approach to address this issue involves collecting biomass, which usually ends up in landfills for incineration without any valorization [17,72]. This poses a significant economic burden, as the removal of large quantities of biomass is difficult and costly [28,72]. For instance, USD120 million was the estimated cost of removing *Sargassum* depositions in the Caribbean in 2015 [73]. In Mexico, USD9.1 million was the cost of restoring the beaches of the Quintana Roo coast [73]. Therefore, the valorization of the collected algae biomass is essential to compensate for the economic impact of its management and removal.

The increasing demand for environmentally friendly agricultural inputs, combined with the need to reduce dependence on synthetic pesticides, has intensified the search for renewable bioactive resources capable of improving crop protection and resilience. In this context, the valorization of invasive macroalgae represents a particularly attractive approach, as it simultaneously addresses biomass overaccumulation and supports the development of sustainable bioproducts with potential applications in disease management and plant defense stimulation.

The macroalgae biomass should be collected before washing ashore to preserve its quality for subsequent valorization, avoid the removal of beach sand, and prevent the spread of invasive algal fragments on the shore [74]. On São Miguel Island, in the Azores Archipelago, 555 metric tons of beached macroalgae were removed and sent to landfills without valorization because of sand and plastic contamination, which rendered the biomass unsuitable for agricultural use [63]. However, the landfill disposal of macroalgal biomass may also generate additional environmental concerns, including methane emissions during anaerobic decomposition and the production of saline or organic-rich leachates that can potentially affect soil and groundwater quality [18,75]. These limitations further highlight the importance of developing sustainable valorization and biomass management strategies.

Macroalgae offshore harvesting helps preserve beach morphology and ecological structure and minimizes the removal of sediment associated with mechanical beach cleaning operations [18,76]. However, the operational feasibility of offshore biomass collection remains highly dependent on local environmental conditions, biomass dispersion patterns, harvesting technologies, and economic constraints [18]. Large-scale offshore harvesting may require specialized vessels, increased fuel consumption, and additional logistical infrastructure, which can significantly increase operational costs and potentially offset some environmental benefits. Consequently, techno-economic assessments are needed to determine whether offshore collection strategies are environmentally and economically viable under different regional scenarios.

Macroalgae are known for their extensive applications. They have historically been used as biofertilizers in agriculture [23]. For instance, macroalgae wrack composed of *Fucus* spp., *Ascophyllum nodosum* and *Pelvetia canaliculata* could mitigate the toxic effects of copper in barley plants, leading to increased leaf length and highlighting its potential as a biostimulant [77]. Macroalgae also possess medicinal properties due to their bioactive secondary metabolites [78]. For example, extracts from *Asparagopsis taxiformis*, an invasive alga, exhibited anticancer, anti-aging, and anticholinesterase activities in vitro [72]. A review by Cunha dos Santos et al. (2024) summarized the dermocosmetic properties of *Sargassum* spp., including the invasive *S. muticum*, highlighting the photoprotective, anti-inflammatory, skin whitening, and antioxidant activities of these algae [79]. Fucoxanthin, a xanthophyll compound, is known for its health-protective effects and is the most prominent pigment in brown algae [80,81]. Macroalgae could also be used as ingredients in aquafeeds, as studies have demonstrated their safety in fish diets [82,83]. Supplementation with macroalgae biomass enhanced the antioxidant defenses in fish. The authors observed a reduction in lipid peroxidation in fish exposed to heat waves [84], and a reduced activity of antioxidant enzymes (e.g., catalase) due to the presence of antioxidant metabolites in the biomass [82,84]. Another potential application is the use of macroalgae for the mitigation of methane emissions in ruminants. In ruminal fermentation experiments, treatment with *Asparagopsis armata* and *Asparagopsis taxiformis* reduced enteric methane production in vitro by 86% and 36%, respectively [85]. These studies highlight the promising potential of macroalgae biomass, although further validation regarding scalability, safety, and economic feasibility is still required. Consequently, the valorization of invasive and beach-cast algae represents a win–win strategy, aiming to mitigate their ecological impact while taking advantage of their bioactive properties.

Agricultural applications represent one of the most immediately feasible valorization routes for invasive macroalgae due to lower purification requirements and compatibility with bulk biomass processing [86]. In contrast, pharmaceutical and cosmetic applications generally require stricter quality control, contaminant monitoring, and compound purification, which may limit the direct use of highly degraded or contaminated beach-cast biomass [87].

## 6. Food Security and Antifungal Properties of Macroalgae

Ensuring food security will require an increase in food production to meet the needs of a globally growing population [88]. However, food loss is a major obstacle. Approximately one-third of the food produced is wasted [89]. Several factors contribute to food loss, including climate change, which reduces crop quality and productivity due to extreme temperatures and soil deterioration [86]. Microbial spoilage is also a major cause of food waste, responsible for 20–40% of global crop losses annually [90]. Approximately 80% of plant diseases are attributed to phytopathogenic fungi [91]. A Top 10 list of phytopathogenic fungi was presented in a review, with *Pyricularia* oryzae (formerly *Magnaporthe oryzae*), *Botrytis cinerea*, and *Puccinia* spp. identified as the most pathogenic [92]. *P. oryzae* is the causal agent of rice blast disease, one of the most destructive diseases affecting global rice production, while *B. cinerea* is responsible for gray mold disease in numerous horticultural and viticultural crops. *Puccinia* species are associated with rust diseases that significantly reduce cereal productivity and quality. Their agricultural relevance makes these pathogens important targets for the development of sustainable antifungal and elicitor-based crop protection strategies [92]. *Botrytis* spp. causes significant economic losses in global agriculture, estimated at USD100 billion annually [93]. *B. cinerea,* specifically, has been detected in vineyards on Madeira Island and poses a threat to local wine production [94], which represents one of the major economic activities of Madeira Island. *B. cinerea* is a necrotrophic fungus that takes advantage of the host cell’s apoptotic processes to reproduce and targets a wide range of plants and fruits [92]. The application of synthetic pesticides is the main strategy to manage infections by phytopathogens, including *Botrytis* spp. [92,95,96]. The continuous use of synthetic chemicals, however, increases soil contamination and constitutes a risk for human health due to toxic residues. Additionally, the resistance mechanisms that these pathogens have developed compromise the efficacy of synthetic pesticides in controlling such infections, further increasing the economic burden of their use [92,97,98]. Given the threat posed by *B. cinerea* to Madeiran viticulture, effective management is of utmost importance. This management should employ sustainable, green alternatives that protect the environment and public health [99].

The use of macroalgae is a sustainable alternative for the management of crops and postharvest fungal infections. Macroalgae are important biostimulants and biofertilizers due to their wide range of secondary metabolites with antimicrobial and elicitor activities [100,101,102]. These metabolites may protect plants through two distinct mechanisms: direct antimicrobial activity and the elicitor-mediated induction of plant defense responses [91]. Antimicrobial activity is the direct action of a compound against a pathogen, such as bacteria and fungi (antibacterial and antifungal activities, respectively), and can occur through different mechanisms that inhibit growth or kill the pathogen directly [103]. Mechanisms of antifungal activity include the disruption of the fungal cell wall and membrane, where antifungal compounds bind to the membrane and form pores, leading to cell death [104,105]. The disruption of fungal membranes is also achieved by inhibiting ergosterol biosynthesis or its activity, as ergosterol is an essential component of the fungal cell membrane [106]. Antifungal compounds can also bind and disrupt membranes of internal organelles, compromising their integrity and cellular functioning [107]. Other antifungal mechanisms include the inhibition of protein synthesis, the suppression of the mitochondrial respiratory chain, and the interference with key fungal enzymatic processes [104,106,108]. These mechanisms vary depending on the chemical nature of the compounds, with phenolics, terpenoids, and polysaccharides acting through distinct biochemical pathways [104,106]. Additionally, macroalgae metabolites have elicitor properties. Plant elicitors are compounds that stimulate plant immunity, typically without direct antimicrobial activity against phytopathogens [109]. Some elicitors, however, also display direct antimicrobial action [110,111]. Plant cells have numerous immune receptors on their surface that recognize pathogen- and microbe-associated molecular patterns (PAMPs and MAMPs, respectively), which are microorganism-derived molecules that trigger defense signaling pathways specific to the type of pathogen detected [112,113,114,115]. The first line of action against pathogens is a hypersensitive response (HR), a mechanism where plants induce rapid cell death at the site of infection to limit the spread of the pathogen to other parts of the plant [110,116]. The initiation of systemic acquired resistance (SAR) or induced systemic resistance (ISR) is a more sophisticated response, mediated by the plant hormones jasmonic acid (JA), ethylene (ET), and salicylic acid (SA) [110,117,118]. The JA, ET, and SA pathways lead to the activation of defensive enzymes such as phenylalanine ammonia-lyase (PAL), tyrosine ammonia-lyase (TAL), chitinase [119,120], and pathogenesis-related (PR) proteins [121]. These pathways also trigger a rapid burst of reactive oxygen species (ROS), which are toxic to phytopathogens [112]. They also lead to phytoalexin biosynthesis, which are chemically diverse secondary metabolites with antimicrobial properties produced de novo by the plant at the site of infection, such as phenolics [121,122]. Another response is the rigidification of plant cell walls due to callose, lignin and suberin deposition [123]. Understanding the interactions between pathogen-derived molecules and plant receptors has led to the discovery of natural or synthetic elicitors that activate the same downstream signaling cascades as those activated by PAMPs and MAMPs [111,115,117,124]. That is, due to their chemical similarity to pathogen-derived molecules, such elicitors interact similarly with plant immune receptors, inducing the same responses [115,117,125]. Examples of natural elicitors that can trigger the same immune responses include carbohydrates, glycoproteins, and lipids [117], which are present in macroalgae.

Macroalgae extracts have shown potential as biopesticides against phytopathogenic fungi, with some results displayed in Table 1. Aqueous formulations of *Ulva rigida* [89], *S. muticum* [95], *Undaria pinnatifida* [126], and *Sargassum fusiforme* [127] showed no antifungal activity in vitro, yet reduced disease severity when applied in infected fruits or plants, suggesting an elicitor action. These extracts induced plant defense mechanisms, including ROS production and enhanced activity of antioxidant enzymes, like superoxide dismutase (SOD) and catalase (CAT) [89,127]. These extracts can also activate the SA and JA pathways through PR1 and IPII gene expression [126]. Sulfated polysaccharides were the major components in the aqueous extracts of *S. muticum* [95] and *U. rigida* [89]. The elicitor properties of algal polysaccharides have been previously addressed, including ulvans, alginates, fucoidans, laminarins, and carrageenan [68,128]. Ulvans, mainly derived from green algae (*Ulva* spp.), are sulfated heteropolysaccharides rich in rhamnose, glucuronic acid, and iduronic acid, and are widely associated with the activation of plant defense responses [129]. Brown algal polysaccharides include alginates, linear copolymers of mannuronic and guluronic acids primarily involved in biostimulant and soil-conditioning effects, as well as fucoidans which are highly sulfated fucose-rich polysaccharides frequently linked to elicitor and antioxidant activities [130]. Laminarins, storage β-glucans from brown algae, are recognized as potent inducers of plant immune responses through the activation of defense signaling pathways [68]. In red algae, carrageenans are sulfated galactans whose biological activity varies according to their sulfation degree and structural type (κ-, ι-, and λ-carrageenans), with several studies reporting their ability to stimulate resistance against phytopathogens [131,132]. For instance, laminarin from *Laminaria digitata* reduced infections by *B. cinerea* and *Plasmopara viticola* in grapevine plants by 55 and 75%, respectively [133], while aqueous ulvan-rich extracts from *Ulva lacinulata* (formerly *Ulva armoricana*) (Chlorophyta) protected bean, grapevine and cucumber plants against powdery mildew pathogens [134]. These findings support the use of aqueous macroalgal extracts with elicitor properties as sustainable alternatives for crop disease management.

However, not all macroalgae species are suitable for crop disease management, as some of them can exceed the threshold levels of heavy metal content allowed for agriculture [135]. Heavy metals of particular concern in macroalgal biomass include arsenic (As), cadmium (Cd), chromium (Cr), mercury (Hg), nickel (Ni), and lead (Pb) whose accumulation depends not only on species-specific uptake capacity but also on environmental conditions and pollution levels in the collection area. Biomass collected near industrialized, urbanized, or heavily polluted coastal regions may present higher contamination risks than biomass harvested from less impacted environments [136,137]. However, this limitation should not be generalized to all invasive macroalgae or collection sites. Heavy metal accumulation is strongly influenced by both species-specific bioaccumulation capacity and local environmental conditions, including anthropogenic pollution sources, coastal hydrodynamics, and sediment characteristics. Consequently, contamination levels may vary substantially among collection sites within the Madeira Archipelago, and site-specific monitoring should be considered before the large-scale agricultural use of recovered biomass. Although *Asparagopsis armata*, *Sargassum muticum*, and *Rugulopteryx okamurae* have all been reported to accumulate trace elements, the available evidence does not currently support the assumption that heavy metal contamination represents a uniform limitation across these species. Instead, contaminant concentrations are likely to depend primarily on environmental exposure and collection location rather than species identity alone.

In a study, the methanolic extracts of *Sargassum dentifolium* (Phaeophyceae), *Gracilaria bursa-pastoris* (formerly *Gracilaria compressa*) (Rhodophyta), and *Ulva lactuca* (Chlorophyta) showed the highest in vitro antifungal activity against *Fusarium oxysporum* (formerly *Fusarium oxysporum f. lycopersici*) among the solvents tested. This higher activity was associated with a greater phenolic content in these extracts, particularly phloroglucinol in *S. dentifolium*, gallic acid in *U. lactuca*, and vanillic acid in *G. bursa-pastoris* [104]. Extracts from *Sargassum cinereum*, *Padina boergesenii*, and *Fucus vesiculosus* have also demonstrated in vitro antifungal activity against *Botrytis cinerea* and other phytopathogenic fungi, as well as promising in vivo efficacy when applied to fruits (Table 1) [95,138]. Phenolic compounds and polysaccharides possess antifungal properties and may be responsible for the fungal growth inhibition observed in these studies [95,139,140]. For instance, purified gallic acid displayed antifungal action against the phytopathogen *Neocosmospora solani* (formerly *Fusarium solani*) [141]. However, some of the studies employed organic solvents for the extraction of compounds with biopesticide activity, which can limit the application of macroalgae extracts for disease management due to the toxicity and environmental risks associated with such solvents [142].

Some species listed in Table 1, such as *A. armata*, *S. muticum*, *R. okamurae*, and *Undaria pinnatifida* which are considered invasive, presented both antifungal and elicitor activity against plant infections [38,39]. Hydroethanolic extracts of *A. armata* inhibited *B. cinerea* growth in vitro by 67.6%, although the aqueous extracts of this alga displayed neither antifungal nor elicitor activity in vivo in pear fruits [95]. In the same study, aqueous extracts of *S. muticum* did not show antifungal activity in vitro, but their application in pear fruits reduced fungal decay by 57.2%, suggesting an elicitor action [95]. Other studies corroborate the elicitor effect of aqueous extracts from *S. muticum* in tomato seedlings and olive tree leaves, which enhanced PAL and TAL activities and stimulated phenolic compounds biosynthesis, which are key mechanisms of plant defense [120,143]. Aqueous extracts of the invasive *R. okamurae* displayed elicitor properties in grapevine plants, reducing disease incidence and severity by 20% and 25%, respectively, against *Plasmopara viticola* [144]. In addition, *R. okamurae*-treated grapevines exhibited increased expression of defense-related genes and signaling pathways, higher production of phytoalexins (phenolic compounds and stilbenes), and the activation of antioxidant enzymes, such as SOD and CAT [121,144]. The class of compounds extracted determines the antifungal and the elicitor activity observed. The presence of phenolic compounds in the hydroethanolic extract of *A. armata* could explain the antifungal effects observed [95]. Furthermore, the aqueous extracts of *S. muticum* were composed of polysaccharides, especially alginates, known to possess elicitor properties [95,120,143]. The higher antifungal activity observed in hydroethanolic extracts compared with aqueous extracts likely reflects differences in extraction selectivity and compound polarity. Hydroethanolic systems are generally more efficient in recovering moderately polar phenolic compounds [145,146] and halogenated metabolites [67,147], which have been associated with membrane disruption and oxidative stress in fungal cells. In contrast, aqueous extracts tend to preferentially recover hydrophilic polysaccharides, which may contribute more strongly to elicitor activity than to direct antifungal effects [68,86]. In the case of *R. okamurae* extracts, the studies did not attribute the elicitor activity to any type of compounds, although previous works have reported the presence of polysaccharides such as alginates, fucoidan and laminarin and phenolic compounds in this alga [148,149,150,151], which may be responsible for the elicitor effects of *R. okamurae* extracts, especially polysaccharides [144]. The use of invasive algae is a promising strategy for managing phytopathogens in crops, as they exhibit antifungal and elicitor activity while contributing to ecosystem preservation through their removal from the shores. Furthermore, the employment of green solvents, such as water and ethanol, is of particular interest, as they meet environmental and food safety requirements, increasing the value of this biomass. These findings highlight the potential of macroalgae-derived extracts as sustainable alternatives to synthetic fungicides. Among the species discussed, *Asparagopsis armata* appears particularly promising for antifungal applications due to its halogenated metabolite profile, whereas brown macroalgae such as *Rugulopteryx okamurae* and *Sargassum muticum* may exhibit stronger elicitor-related activity because of their higher content of fucoidans and phlorotannins [152]. However, direct comparisons remain difficult due to differences in extraction methodologies, target pathogens, and bioassay conditions among studies.

Phenolic compounds, particularly phlorotannins from brown macroalgae, are frequently associated with direct antifungal effects due to their ability to interfere with membrane integrity and oxidative balance [153]. Conversely, sulfated polysaccharides such as ulvans, carrageenans, and fucoidans are more commonly linked to elicitor activity through the activation of plant defense pathways involving salicylic acid and jasmonic acid signaling [68]. Terpenoids and halogenated compounds, especially from red macroalgae, may additionally contribute to antifungal activity through membrane destabilization and the inhibition of fungal metabolic processes [67].

Importantly, direct antifungal activity and elicitor-mediated plant protection should not be considered equivalent mechanisms, as several macroalgal extracts lacking strong in vitro antifungal effects may still induce significant resistance responses in planta.

**Table 1:** Overview of invasive and non-indigenous macroalgae reported in the literature, including their main bioactive compounds and associated biological activities relevant to plant protection. N.A.—Not Applicable.

Macroalgae	Group	Target Pathogen	Plant/Fruit	Extract	Activity Classification	Results	Ref.
*Ulva rigida*	Green	*Botrytis cinerea*	Postharvest grapes	Aqueous solution/submersion	Elicitor	In vitro—No antifungal activity (Agar plate) In vivo—Decrease in incidence and decay area by 43 and 41%. Induction of defense mechanisms: ROS production, increase in SOD, CAT, and chitinase activity.	[89]
*Asparagopsis armata* *Sargassum muticum* *Fucus vesiculosus*	Red Brown Brown	*Botrytis cinerea*	Pear	Hydroethanolic Aqueous Aqueous Aqueous Aqueous Aqueous	Antifungal Elicitor Dual activity	In vitro: Mycelial growth inhibition by 68% (Agar plate) In vivo: No antifungal activity In vitro: No antifungal activity In vivo: Decrease in decay area by 57% In vitro*:* Mycelial growth inhibition 60% In vivo*:* Decrease in decay area by 31%	[95]
*Sargassum cinereum* *Padina boergesenii*	Brown	*Botrytis cinerea*	Strawberry	Organic Methanolic Organic Methanolic	Dual activity Dual activity	In vitro: Mycelial growth inhibition In vivo: Decrease in decay area In vitro: Mycelial growth inhibition In vivo: Decrease in decay area	[138]
*Sargassum fusiforme*	Brown	*Botrytis cinerea*	Tomato plant	Aqueous/FS	Elicitor	In vitro*:* No antifungal activity In vivo*:* Reduced disease severity by 37%. ROS production	[127]
*Laminaria digitata*	Brown	*Botrytis cinerea*	Grapevine plant	Laminarin	Elicitor	In vivo*:* Decrease in lesion area by 55%	[133]
*Ecklonia* sp. *Jania* sp.	Brown Red	*Botrytis cinerea*	Strawberry	Polysaccharides from aqueous extracts	Elicitor	In vivo*:* Reduction in fruit infected area upon the application of polysaccharides, with better results for *Jania* sp.	[139]
*Rugulopteryx okamurae* and *Ulva ohnoi*	Brown Green	N.A.	Grapevine plant (greenhouse)	Aqueous/FS	Elicitor	In vivo: Elicitor activity, including activation of defense genes PR10, PAL, STS48 and GST1. Increase in secondary metabolites production (polyphenols and stilbenes). Increase in antioxidant enzyme activity	[121]
*Undaria pinnatifida*	Brown	*Phytophthora infestans*	N.A.	Aqueous/FS	Elicitor	In vitro: No antifungal activity for the 10% extract In vivo: Decrease in decay area by 60%. Activation of defense mechanisms (SA and JA pathways)	[126]
*Sargassum dentifolium*	Brown	*Fusarium oxysporum*	Tomato plant (greenhouse)	Methanolic Dry powder	Dual activity	In vitro: Mycelial growth inhibition (Agar plate) In vivo: Decrease in decay area by 41%	[104]
*Bifurcaria bifurcata*	Brown	N.A.	Tomato plant (greenhouse)	Aqueous saccharide solution	Elicitor	In vivo—elicitor activity, including PAL activation and phenolic compounds biosynthesis	[154]
*Ascophyllum nodosum*	Brown	N.A.	Arugula plant (*Eruca sativa*)	Aqueous	Dual activity	In vivo—elicitor activity, including PAL and PPO activation, phenolic compounds biosynthesis, and activation of SOD and CAT enzymes	[155]
*Rugulopteryx okamurae*	Brown	*Plasmopara viticola*	Grapevine plant (*Vitis vinifera* L. cv)	Aqueous	Dual activity	In vivo—elicitor activity, including activation of defense genes, phenolic compounds biosynthesis and SOD, APX, and GR antioxidant enzymes. Reduction in disease incidence and severity by 20 and 25%	[144]
*Ascophyllum nodosum*	Brown	*Plasmopara viticola*	Grapevine plants (greenhouse)	Aqueous /FS	Dual activity	In vitro—Spore count reduction by 35% (leaf disk assay) In vivo—Disease incidence reduction (40.00%) compared to control (70%)	[156]
*Laminaria digitata*	Brown	*Plasmopara viticola*	Grapevine plant (greenhouse)	Laminarin	Elicitor	In vivo—Decrease in decay area by 75%	[133]
*Codium decorticatum*	Green	N.A.	Tomato seedlings	Aqueous (FS and internodal injection)	Elicitor	In vivo—elicitor activity, including PAL activation, and induction of biosynthesis of phenolic compounds and lignin	[157]
*Codium tomentosum*	Green	N.A.	Pear	Aqueous	Antimicrobial/Postharvest protection	In vivo—Shelf-life extension observed in fruits: Less color change. Inhibition of yeast and mold development	[142]
*Sargassum muticum*	Brown	N.A.	Tomato seedlings	Aqueous alginate solutions/FS	Elicitor	In vivo—elicitor activity, including PAL activation, biosynthesis of phenolic compounds and lignin	[143]
*Sargassum muticum*	Brown	N.A.	Olive tree leaves	Polysaccharide aqueous solutions	Elicitor	In vitro (leaf disks)—elicitor activity: PAL and TAL activation, and phenolic compounds and lignin biosynthesis	[120]

The studies summarized in Table 1 highlight that elicitor activity is more frequently reported than direct antifungal activity among macroalgal extracts. While some extracts exhibit dual activity, direct inhibition of phytopathogens appears to be more commonly associated with phenolic- and terpenoid-rich extracts, whereas polysaccharide-rich fractions are predominantly linked to plant defense induction.

A comparative analysis of the available studies reveals important differences among macroalgae groups and extract types. Red macroalgae, particularly *Asparagopsis armata*, generally exhibited stronger direct antifungal activity, which is likely related to their high content of halogenated secondary metabolites. *Asparagopsis armata* is particularly rich in halogenated secondary metabolites, including brominated and iodinated compounds, which have been associated with strong antimicrobial and antifungal activities through membrane disruption and interference with cellular metabolic processes [67,158]. This biochemical profile may explain the higher direct antifungal activity reported for hydroethanolic extracts of *A. armata* compared with several other macroalgae species.

In contrast, brown macroalgae such as *Laminaria digitata*, *Ascophyllum nodosum*, *Sargassum muticum*, and *Rugulopteryx okamurae* more frequently displayed elicitor-related activity through the activation of plant defense pathways, including PAL activity, phenolic compound biosynthesis, and salicylic acid- and jasmonic acid-mediated responses, possibly due to the higher abundance of phlorotannins, alginates, fucoidans, and other sulfated polysaccharides, although the specific compounds responsible for plant defense induction remain largely unidentified [159,160]. Green macroalgae, represented mainly by *Ulva* spp. and *Codium* spp., also showed promising elicitor properties, although evidence of direct antifungal activity remains limited [157,161].

Regarding extraction strategies, hydroethanolic and methanolic extracts generally produced higher levels of direct antifungal activity than aqueous extracts, likely due to their greater capacity to recover moderately polar phenolics, terpenoids, and halogenated metabolites. Conversely, aqueous extracts and polysaccharide-rich fractions were more commonly associated with elicitor activity, suggesting that extraction selectivity plays a major role in determining the biological response observed.

## 7. Green Extraction Technologies

The current strategy for the recovery of natural, value-added compounds involves the use of petrochemical and volatile organic solvents (VOCs), most of which are flammable and harmful to both human health and the environment. Therefore, greener and more sustainable strategies are being prioritized to address safety concerns [162,163], including the use of green solvents such as ethanol, methyl esters of fatty acids and glycerol, among others [163]. Another alternative to VOCs is the use of deep eutectic solvents (DES). Chemically, these solvents consist of mixtures of Brønsted–Lowry or Lewis acids and bases with different types of cationic and anionic groups [164]. These components establish strong hydrogen bonds, which lead to a depression of the melting point of the final mixture compared to that of the pure compounds [165]. DES are prepared by mixing at least one hydrogen-bond acceptor (HBA) and one hydrogen-bond donor (HBD), generally using the heating and stirring method at temperatures of 50 to 100 °C until a homogeneous liquid is formed [166,167]. DES were first popularized by Abbott et al. (2003) [168], who demonstrated that mixtures such as choline chloride and urea form stable eutectic liquids with unique solvent properties, with melting points much lower than those of the separate components. Various HBAs and HBDs can be used for the preparation of eutectic mixtures. Natural deep eutectic solvents (NADES) were first proposed by Choi et al. (2011) and refer to DES composed of primary metabolites such as amino acids, sugars, vitamins, and natural carboxylic acids, among others [169,170]. The most common HBA component to prepare NADES is choline chloride (ChCl), as it is environmentally friendly, inexpensive and non-toxic to humans [164]. Choline is a constituent of vitamin B and is widely produced worldwide as a nutritional supplement [171]. Common safe HBDs include urea and glycerol, which have been mixed with ChCl to prepare NADES [164,168]. Urea has been utilized as a fertilizer in agriculture [171]. DES and NADES offer several advantages, including simple and cost-effective synthesis, low volatility, low vapor pressure, non-flammability, and chemical and thermal stability [172,173,174,175]. Furthermore, the physicochemical properties of DES solvents can be easily tuned (Table 2) to extract specific compounds from a given matrix [176]. NADES solvents represent a green, sustainable alternative to VOCs for extracting value-added compounds in future applications.

Despite all the advantages related to DES and NADES systems, no studies have explored the direct use of NADES extracts as biopesticides, since most studies involving DES have focused on analytical chemistry, extraction technologies, and therapeutic agents [177,178]. Recent evidence suggests that certain NADES formulations may possess intrinsic antimicrobial and antifungal activities, especially those based on phenolic and terpenoid constituents such as thymol and menthol, indicating that the biological activity of NADES-based extracts may result not only from the extracted metabolites but also from synergistic interactions between the solvent system and the target compounds [179]. This constitutes an important research gap that hinders the application of such systems in the food industry to combat microbial infections. For instance, studies on the behavior of DES on fruit surfaces, including adhesion and diffusion properties as well as a comprehensive evaluation of their toxicity, are not available [177].

However, several studies have used NADES for the recovery of macroalgal compounds with known antifungal and elicitor activities, including phenolics and polysaccharides (Table 3). Direct comparison between studies is difficult, as several factors influence the extraction of bioactive compounds from macroalgal biomass. The extraction yields vary depending on the algal species and the origin of the biomass [180]. For instance, brown algae generally contain higher levels of phenolic compounds and polysaccharides than green and red algae [181]. Additionally, the geographical location and climatic conditions influence the concentration of bioactive compounds in algae [180]. The season of collection is also crucial, as the concentration of secondary metabolites in algae tends to vary throughout the year [182,183]. Extraction conditions are another important factor, as they directly affect recovery yields. However, they are hardly reproducible. These conditions, such as temperature, can fluctuate throughout the process, and different types of equipment are usually used even for the same extraction method. Various extraction methods can be employed, such as ultrasound-assisted extraction (UAE), microwave-assisted extraction (MAE), and maceration [184]. Additionally, quantification methods, such as total phenolic assays, can influence the results due to the use of different standards across studies and the presence of contaminants in the extracts that may interfere with the assay [180]. All these factors contribute to a loss in reproducibility, as they affect extraction yields and therefore make direct comparison between studies inaccurate.

Various parameters affect the performance of NADES in the extraction of bioactive compounds. The composition of the eutectic mixture, including the types of HBA and HBD and water content, affects the solvent’s polarity and its capacity to solubilize different types of compounds [185]. The composition also affects the thermal stability and viscosity of NADES [185,186]. In a previous study, lactic acid-based NADES improved extraction yields of phenolics at higher contents in lactic acid probably due to polarity effects [176]. Some NADES based on alkanol amines and polyols showed initial thermal degradation from 80 °C, which varied depending on the nature of the components as well as the proportion of mixture [186]. The combination of choline chloride: ethylene glycol (1:2) exhibits relatively low viscosity, whereas ChCl-sugar and ChCl-metal-based NADES show significantly higher viscosities [172]. Aqueous NADES exhibited superior performance compared to pure NADES for the extraction of phenolic compounds from brown algae *Sargassum filipendula*, *Fucus vesiculosus* and *Ascophyllum nodosum* [186,187]. Water helps weaken the hydrogen bonds between the HBA and HBD, reducing viscosity and facilitating mass transfer, improving extraction yields [188,189,190]. However, excessive water content destabilizes the eutectic mixture by disrupting the hydrogen bonds between its constituents [188]. External factors such as temperature and pressure also influence the extraction performance of NADES. The extraction yield of polysaccharides from *Saccharina japonica* increased with temperature, particularly in the case of alginate and fucoidan [165]. Higher temperatures reduce the viscosity of NADES, thereby improving mass transfer from the biomass [165,190]. High temperatures, however, may lead to the thermal degradation of NADES and the target compounds [170,184,186]. The composition of NADES determines their performance in extracting bioactive compounds as well as their stability under different temperature and pressure conditions. The thermal stability of NADES should be evaluated before the extraction process, which can be assessed using thermogravimetric analysis [171].

NADES can offer increased extraction yields of phenolic compounds and polysaccharides compared to conventional organic and aqueous solvents. A study reported a total phenolic content (TPC) value of 15.30 mg GAE.g^−1^ dw from *S. muticum* using a eutectic mixture of proline:1,2-Butanediol (1:3) with 30% water [180]. In another study, from the same researchers, they reported a TPC value of 6.30 mg GAE.g^−1^ dw (2099 mg GAE.L^−1^) for the same alga using a eutectic mixture of lactic acid to fructose of 7:1 (LA:Fru 7:1) with 25% water and solid-to-liquid (S/L) ratio of 1:3 [176]. These TPC values were higher than those obtained with H_2_O and EtOH/H_2_O (70:30) rendering values of 5.64 and 3.70 mg GAE.g^−1^ dw, respectively [176,180]. Additionally, the eutectic mixture of proline:1,2-butanediol (1:4) yielded 20.61 mg of quercetin per gram (total flavonoid content) from *S. muticum*, which is approximately twice to three times higher than the values obtained with H_2_O and EtOH:H_2_O (70:30), respectively [180]. Another study investigated the potential of DES solvents based on alcohol amines for the extraction of phlorotannin and pigments from *Sargassum filipendula*. The authors noted enhanced yields of TPC and total phlorotannin contents using DES and aqueous DES compared to conventional solvents, such as EtOH:H_2_O, H_2_O, NaOH 1%, MeOH and CHCl_3_ [186]. Another study reported polysaccharide extraction yields of 11.31 and 13.52% with a eutectic mixture of choline chloride-1,2-propanediol (ChCl:12PDO, 1:2) and water, respectively. The extraction methods, however, were different for each solvent: ultrasound-assisted extraction for 30 min was used with DES, while heating was used with hot water during 2 h (decoction) [191]. The composition of NADES can be tailored to extract specific compounds and enhance the results compared to conventional solvents. Both the HBA and HBD and the content of water govern NADES interactions with the target compounds [172,192]. The available information highlights NADES as a promising alternative to conventional organic solvents for the extraction of valuable compounds, such as phenolics and polysaccharides. Despite their promising properties, DES/NADES systems still face important limitations regarding large-scale implementation (Table 2). High viscosity may hinder mass transfer and increase energy requirements during processing, while solvent recovery and downstream purification remain challenging compared to conventional volatile solvents. In addition, the regulatory status and ecotoxicological safety of residual DES/NADES components in agricultural formulations require further investigation before industrial application.

**Table 2:** Main differences between extraction systems used to purify main compounds from macroalgae.

Extraction System	Advantages	Limitations	Most Recovered Compounds
Water	Safe, low cost	Low selectivity	Polysaccharides
Hydroethanolic	Good phenolic recovery	VOC use	Phenolics
DES/NADES	Tunable polarity	High viscosity	Phenolics/pigments
Supercritical CO_2_	Selective, solvent-free	Expensive	Lipophilic compounds

**Table 3:** Extraction methods used for macroalgae biomass, with emphasis on green technologies such as deep eutectic solvents (DES/NADES), including operational conditions and target compounds.

Macroalgae	Group	DES (HBA/HBD)	Extraction Method	Extraction Yield	Reference
*Sargassum* spp.	Brown	ChCl:Urea (1:2), 30% water ChCl:Glycerol (1:2), 30% water	UAE, 1 h, 70 °C	Fucoidan: 0.0025 g.g ^−1^ dw Alginate: 0.4103 g.g ^−1^ dw	[170]
*Sargassum muticum* *Gelidium corneum*	Brown Red	LA:Fru (7:1), 25% water, 1:3 S/L LA:Fru (5:1), 25% water	Maceration, 2 h, RT	TPC: 2099.0 mg GAE.L^−1^ TPC: 408.4 mg GAE.L^−1^	[176]
*Sargassum filipendula*	Brown	DMAE:BzOH (1.30:1), 30% water, S/L 0.17 DMAE:13PDO (1.83:1), 30% water, S/L 0.14	Stirring, 2 h, 120 °C	TPC: 78.15 mg GAE.g^−1^ Phlorotannins: 110.64 mg PEG.g^−1^ TPC: 72.89 mg GAE.g^−1^ Phlorotannins: 21.57 mg PEG.g^−1^	[186]
*Sargassum horneri*	Brown	Hot water ChCl:12PDO (1:2), 30% water, S/L 1:30	Decoction, 2 h, 95 °C UAE, 30 min, 70 °C	Polysaccharides: 13.52% yield Polysaccharides: 11.31% yield	[191]
*Sargassum muticum*	Brown	Pro:1,2-But (1:3), 30% water, S/L 1:10 Pro:PPG (1:4), 30% water, S/L 1:10	Maceration, 100 min, 60 °C	TPC: 15.30 mg GAE.g^−1^ dw TPC: 15.26 mg GAE.g^−1^ dw	[180]
*Saccharina japonica*	Brown	ChCl:Glycerol (1:2), 70% water, L/S 36.81	Subcritical water, 150 °C. 19.55 bar	Alginate: 28.12% yield Fucoidan: 14.63% yield	[165]
*Fucus vesiculosus* *Ascophyllum nodosum*	Brown	ChCl:LA (1:1–1:3), 50–70% water, S/L 1:5	Maceration, 2 h, 50 °C	Phlorotannins: ~69% yield Phlorotannins: ~72% yield	[187]

Comparison among extraction technologies indicates that conventional organic solvents frequently achieve higher extraction yields of lipophilic antifungal compounds; however, their environmental impact and toxicity remain important limitations. DES and NADES systems offer a more sustainable alternative and have demonstrated promising extraction efficiency for phenolics, flavonoids, and polysaccharides while reducing the use of hazardous solvents. Nevertheless, direct comparisons among extraction systems remain scarce, making it difficult to establish whether DES/NADES consistently outperform conventional solvents across different macroalgae species and target bioactivities. Future studies should therefore focus on standardized comparative evaluations that simultaneously assess extraction yield, compound selectivity, antifungal efficacy, solvent recovery, and economic feasibility.

## 8. Challenges and Future Perspectives

Despite the growing interest in the valorization of non-indigenous and beach-cast macroalgae, several limitations still hinder their large-scale implementation. Biomass availability and composition are highly variable and depend on environmental conditions, seasonal fluctuations, life stage, and decomposition state, which may significantly affect the yield and consistency of bioactive compounds. In addition, contamination by sand, plastics, salts, or heavy metals can compromise biomass quality and increase processing costs. Standardized protocols for biomass collection, storage, and pre-treatment are therefore essential to improve reproducibility and scalability.

Green extraction technologies such as DES and NADES have emerged as promising alternatives to conventional solvents due to their tunable physicochemical properties and lower environmental impact. They also have demonstrated promising extraction efficiency for phenolics, polysaccharides, and other bioactive compounds. However, challenges related to solvent viscosity, extraction optimization, downstream purification, solvent recovery, and compound stability still limit industrial applicability. Furthermore, most studies remain restricted to laboratory-scale evaluations, with limited techno-economic assessments and field validation of macroalgae-derived products. In particular, the improved extraction performance achieved by some DES/NADES formulations must be balanced against downstream processing requirements, including solvent recovery, compound purification, and product formulation. The high viscosity of many DES/NADES systems may increase mass transfer limitations and processing costs, while the separation of target compounds from the extraction medium can be more complex than in conventional solvent-based processes. Consequently, the economic feasibility of DES/NADES extraction will depend not only on extraction yield but also on the development of efficient solvent recycling strategies and cost-effective downstream processing technologies. Future studies should therefore combine extraction efficiency assessments with techno-economic analyses and life-cycle assessments to determine whether the environmental advantages of DES/NADES systems can be translated into economically viable industrial processes.

Regulatory uncertainty regarding the use of non-indigenous biomass also represents an important barrier for commercialization. Future efforts should therefore focus on developing harmonized regulatory frameworks, scalable extraction strategies, and integrated biorefinery approaches capable of converting invasive biomass into multiple value-added products. Although the valorization of invasive and beach-cast macroalgae aligns with circular bioeconomy principles, additional research is still required to demonstrate the long-term environmental, economic, and technical feasibility of these approaches under real-world conditions.

## 9. Conclusions

Non-indigenous and beach-cast macroalgae represent significant ecological and economic challenges due to their capacity to alter ecosystems and negatively affect coastal activities such as fisheries and tourism. At the same time, their large biomass availability and rich biochemical composition make them attractive candidates for biomass valorization within circular bioeconomy frameworks. Recent studies have highlighted the potential of these macroalgae as sources of bioactive compounds, including phenolics, terpenoids, and sulfated polysaccharides, with reported antifungal and elicitor-related activities relevant for sustainable crop protection.

Green extraction technologies, particularly Deep Eutectic Solvents (DES) and Natural Deep Eutectic Solvents (NADES), have emerged as promising alternatives to conventional organic solvents due to their tunable physicochemical properties and lower environmental impact. Nevertheless, the available evidence remains limited for invasive macroalgae species, and direct comparisons among extraction systems are often complicated by differences in biomass composition, extraction conditions, and bioassay methodologies. Furthermore, most studies remain restricted to laboratory-scale evaluations.

Several unresolved challenges still constrain the large-scale implementation of invasive macroalgae valorization strategies. Biomass heterogeneity, seasonal variability, contamination by sand, plastics, or heavy metals, and the instability of certain bioactive compounds may significantly affect extraction performance and product standardization. In addition, DES/NADES systems still face important technical and economic limitations related to solvent viscosity, downstream purification, solvent recovery, scalability, and regulatory acceptance for agricultural applications. The ecotoxicological implications of residual solvent components in formulated products also require further investigation.

Future research should prioritize the standardization of biomass collection and processing protocols, optimization of extraction parameters according to target compounds, and the development of scalable and economically viable extraction systems. More comprehensive metabolomic profiling and mode-of-action studies are needed to better establish the relationships between macroalgal metabolites and their antifungal or elicitor activities. Importantly, long-term greenhouse and field trials remain essential to validate the efficacy, safety, and consistency of macroalgae-derived products under realistic agricultural conditions.

Overall, invasive and beach-cast macroalgae may represent a promising but still underdeveloped resource for sustainable bioproduct development. Advancing their practical application will require interdisciplinary collaboration among researchers, industry, and regulatory authorities to ensure that environmental, economic, and technological limitations are adequately addressed before large-scale commercialization can be achieved.

## Data Availability

Data are available from the corresponding author upon reasonable request.

## Abbreviations

The following abbreviations are used in this manuscript:

NIS	Non-Indigenous Species
DES	Deep Eutectic Solvents
NADES	Natural Deep Eutectic Solvents
HBA	Hydrogen-Bond Acceptor
HBD	Hydrogen-Bond Donor
VOCs	Volatile Organic Compounds
UAE	Ultrasound-Assisted Extraction
MAE	Microwave-Assisted Extraction
TPC	Total Phenolic Content
TFC	Total Flavonoid Content
GAE	Gallic Acid Equivalents
PEG	Phloroglucinol Equivalents
dw	Dry Weight
S/L	Solid-to-Liquid Ratio
RT	Room Temperature
FS	Foliar Spray
ROS	Reactive Oxygen Species
SOD	Superoxide Dismutase
CAT	Catalase
APX	Ascorbate Peroxidase
GR	Glutathione Reductase
PAL	Phenylalanine Ammonia-Lyase
TAL	Tyrosine Ammonia-Lyase
PPO	Polyphenol Oxidase
PR	Pathogenesis-Related
SA	Salicylic Acid
JA	Jasmonic Acid
PR1	Pathogenesis-Related Protein 1
IPII	Proteinase Inhibitor II
GST1	Glutathione S-Transferase 1
STS48	Stilbene Synthase 48
EU	European Union
MPA	Marine Protected Area
FAO	Food and Agriculture Organization
FEDER	European Regional Development Fund
FCT	Foundation for Science and Technology
ChCl	Choline Chloride
LA	Lactic Acid
Fru	Fructose
Pro	Proline
PPG	Propylene Glycol
12PDO	1,2-Propanediol
13PDO	1,3-Propanediol
DMAE	Dimethylethanolamine
BzOH	Benzyl Alcohol
